# Clinical Outcomes for Sexual and Gender Minority Adolescents in a Dialectical Behaviour Therapy Programme

**DOI:** 10.1017/S135246582400016X

**Published:** 2024-04-08

**Authors:** J. Camp, G. Durante, A. Cooper, P. Smith, K.A. Rimes

**Affiliations:** 1 National & Specialist CAMHS, DBT Service, South London & Maudsley NHS Foundation Trust, Micheal Rutter Centre, Maudsley Hospital, London, SE5 8AZ; 2 Department of Psychology, Institute of Psychiatry, Psychology, & Neuroscience, King’s College London, De Crespigny Park, London, SE5 8AB; 3 Department of Psychology, Goldsmiths University of London, Lewisham Way, London, SE14 6NW

**Keywords:** LGBTQ+, Adolescents, Self-injury, Emotion Dysregulation, DBT

## Abstract

**Background:**

Sexuality and gender minoritised (SGM) adolescents are at increased risk of self-injury and suicide, and experience barriers to accessing mental health support. Dialectical Behaviour Therapy (DBT) is an effective treatment for self-injury and emotion dysregulation in adolescent populations, but few studies have published outcomes of DBT for SGM young people.

**Aims:**

This study aimed to investigate treatment outcomes and completion for SGM adolescents and their cisgender and heterosexual peers, in the National & Specialist CAMHS, DBT service (UK).

**Methods:**

Treatment completion, opting out before and during treatment were examined for sexual and gender identity groups, as well as changes by the end of treatment in emotion dysregulation, self-injury, inpatient bed days, emergency department attendances, and borderline personality disorder, depression, and anxiety symptoms.

**Results:**

SGM adolescents were over-represented in this service, even after considering their increased risk for self-injury. No statistically significant differences were found for treatment completion between the sexual orientation and gender identity groups although there were patterns indicating possible lower treatment uptake and completion that warrant further investigation. Clinical outcomes for treatment-completers showed improvement by the end of DBT for each group, with few exceptions.

**Discussion:**

These results are from relatively small subsamples, and it was not possible to separate by sex assigned at birth. Findings should be treated tentatively and as early indications of effect sizes to inform future studies. This study suggests that DBT could be a useful treatment for SGM adolescents in a highly specialist treatment setting.

## Introduction

Suicidal and non-suicidal self-injury (NSSI) in adolescent populations is a growing issue and is significantly associated with death by suicide (Bould, Mars, Moran, Biddle, & Gunnell, 2019; Cheung, McKeown, Schneider, & Shah, 2020; Geulayov et al., 2018; Heron, 2016; Hawton et al., 2020; McManus et al., 2019). Sexuality and gender minoritised (SGM) adolescents are 3 times more likely to engage in self-injurious behaviours and 1.5 to 7 times more likely to experience suicidal thoughts and attempt suicide compared to cisgender and heterosexual young people (Butler et al., 2019; Haas et al., 2011; Kapatais, Williams, & Townsend, 2022; King et al., 2008; Liu et al., 2019; Raifman et al., 2020). Sexual minorities are broadly defined as those who experience sexual and/or romantic attractions congruent with, or identify as, lesbian, gay, bisexual, pansexual, asexual, queer, and other minoritised orientations. Gender minorities are defined as those whose gender identity is different from their sex assigned at birth (transgender), is outside of the traditional gender binary (non-binary), and other gender-diverse groups.

The higher prevalence of suicidal and NSSI in SGM adolescents is congruent with a higher prevalence of associated mental health difficulties, including emerging symptoms of borderline personality disorder (BPD), depression, and anxiety disorders (Liu et al., 2019; Rodriguez-Seijas, Morgan, & Zimmerman, 2021; Ross et al., 2018; Russel & Fish, 2016). Mental health disparities in SGM populations are thought to be explained by exposure to prejudice, discrimination, and victimisation associated with their minoritised identity in societies that privilege cisgender (i.e. gender is broadly the same as sex assigned at birth) and heterosexual norms, and that invalidate SGM culture (i.e. cis-heteronormativity and cis-heterosexism; Batchmann & Gooch, 2017; Hendricks & Testa, 2012; Hubbard, 2021; Meyer, 2003). Minority stress theory suggests that the additional minority-related stressors, alongside everyday stressors, cause an accumulation of stress such that it overwhelms typical resilience and coping mechanisms for individuals (Hendricks & Testa, 2012; Meyer, 2003). Building on minority stress theory, the psychological mediation framework suggests that the mechanisms by which these stressors mediate mental health outcomes include increased likelihood of psychological processes typically implicated in mental health difficulties, such as emotion dysregulation (Hatzenbuehler, 2009), which are also shown to be elevated in SGM populations (Kapatais et al., 2022).

Despite the increased mental health needs of SGM youth, these groups are shown to experience significant barriers to accessing health care (Dunbar, 2017; McDermott, 2016; McDermott, Hughes, & Rawlings, 2018; Williams and Chapman, 2011). Moreover, certain SGM groups report poor experiences in services (Beard et al., 2017; Hubbard, 2021; Rimes, Ion, Wingrove, & Carter, 2019; Williams and Chapman, 2011). Reviews of the literature suggest that SGM youth may experience poor quality of care due to clinician-held stigma and lack of awareness of the unique needs of this population, as well as from a fear of being judged or accidently ‘outed’ to others (Hafeez et al., 2017; Pattinson et al., 2021). This may also be impacted by the lack of SGM-specific mental health service provision and the over-reliance on the charitable sector to provide this support, which may be more vulnerable to volatility in funding and resource (Pattingson et al., 2021). Very few effectiveness and efficacy studies collect data or stratify samples by sexual orientation and gender identity, and routine clinical services rarely report such comparative outcomes, therefore little is currently known about the effectiveness of interventions for SGM groups (Harned, Coyle, & Garcia, 2022; Pachankis, 2018).

Dialectical Behaviour Therapy (DBT) is a third-wave cognitive-behavioural therapy developed for individuals experiencing high levels of self-injury, suicidal ideation, and emotion dysregulation (Linehan, 1993). DBT is an effective intervention for suicidal and NSSI behaviours in young people (Johnstone, Marshall, & McIntosh, 2021; Kothgassner et al., 2021) and is recommended by the National Institute for Health and Care Excellence (2022; UK) and National Institute of Mental Health (2022; USA) for the treatment of self-injury and emotion dysregulation in adolescent populations.

The DBT model is thought to have useful applications for distress associated with SGM experiences (Camp, 2023; Camp, Morris, et al., 2023; Cohen, Norona, Yadavia, & Borsari, 2021; Penta et al., 2022; Skerven, Whicker, & LeMaire, 2019; Sloan, Berke, & Shipherd, 2017). One reason for this is that DBT is principle-driven, which allows flexibility to integrate SGM-specific issues into the treatment as needed. In addition, Linehan’s (1993) transactional biosocial model, which underpins the understanding of how difficulties develop, is thought to be complementary to minority stress theories. The “biological” aspect of this model suggests that a person may have a societally-minoritised (e.g. gender diversity from a young age; Grove & Crowell, 2019) or temperamental (e.g. emotion sensitivity) difference, which makes it more likely that developmentally-important others and society will invalidate their experiences. An invalidating developmental environment (the “social” part of the model) subtly and explicitly teaches the person that their experiences related to their differences and internal world are invalid and/or that complex problems are easily solved (Grove & Crowell, 2019; Linehan, 1993). Transactions between potential vulnerabilities and invalidating environments amplify and change dynamically over time, resulting in an individual not learning how to understand and label their emotions, thus learning to mistrust their experiences; internalising invalidating messages (self-invalidation); and under-estimating the complexity of solving life problems. This often leads to a lack of problem-solving skills, difficulties tolerating distress, and impairment in setting reasonable goals and expectation. This is thought to cause a skills deficit or breakdown in the context of managing complex life stressors (such as those experienced by SGM individuals), contributing to the development of emotion dysregulation (Grove & Crowell, 2019; Linehan, 1993). Difficulties regulating emotions likely leads to dysregulation in other aspects of life (e.g. identity confusion, relationships difficulties, and impulsive behaviours; Linehan, 1993). Congruent with the minority stress and psychological mediation models, the biosocial model may be a useful way to explain how SGM’s experiences and the internalisation of invalidation may result in the development of emotion dysregulation, which may contribute to psychological distress and ‘behavioural dysregulation’, such as self-injury (Cohen et al., 2021; Sloan et al., 2017).

Another aspect of DBT thought to be applicable to SGM groups is the focus on the transdiagnostic construct of emotion dysregulation as the core treatment target, which is considered an important mediating factor between minority stress and mental health outcomes in the psychological mediation framework (Hatzenbuehler, 2009). Finally, the use of validation, a dialectical world view, and teaching skills that are generalisable to varied life problems, makes DBT a potentially ideal candidate for supporting SGM young people experiencing psychological distress and discrimination (Cohen et al., 2021; Penta et al., 2022; Skerven et al., 2019; Sloan et al., 2017). This should not be seen to imply that it is incumbent on minoritised individuals to take responsibility for managing societal oppression. However, while such oppression exists, it is considered that DBT may offer an effective way to support SGM youth to understand how difficulties develop in relation to their minoritised identity and support them to cope and thrive, given that societal change is slow. It should also be balanced with empowering minoritised individuals to advocate for their needs and societal change.

Some authors have considered how to adapt the DBT model for SGM-specific needs in adult samples. Cohen et al. (2021) and Skerven et al. (2021) have both developed an adaptation to DBT skills training to include skills specific to SGM minority stressors and tailored SGM-specific teaching examples. These adaptations have been piloted with a small number of SGM adult veterans in the USA with promising outcomes. In a similar vein, a number of authors also suggest ways that the DBT model and principles, more generally, can be applied to SGM populations (Camp, 2023; Camp, Morris, et al., 2023; Pantalone et al., 2019; Skerven et al., 2019; Sloan et al., 2017; Tilley et al., 2022). However, these recommendations have generally not been empirically tested nor has consideration been given to specific application of DBT for SGM adolescent populations. One study has investigated outcomes of a DBT-informed intervention (interpersonal effectiveness skills), embedded within a wider cognitive-behavioural intervention, in an adult partial-hospital setting where the sample was stratified by those who identified as heterosexual and sexual minorities (Beard et al., 2017). The study found no significant differences between groups in anxiety and depression symptoms, and substance use at the end of the intervention. However, they found that the bisexual group, compared to the heterosexual and gay/lesbian group, had higher self-injury and suicidal thoughts, and worse perceptions of care at the end of treatment. This finding is similar to those observed in outcome research for adult CBT services in the UK, where bisexual (but not gay) men had worse outcomes compared to heterosexual men; for women both lesbian and bisexual patients had poorer outcomes than heterosexual women (Rimes, Ion, et al., 2019).

Only one known study has investigated equality of outcomes between sexuality minoritised and heterosexual adolescents within a non-adapted 18-week DBT programme for adolescents (Poon et al., 2022). No significant differences were found in changes in depression, anxiety, BPD symptoms, emotion dysregulation, and adaptive and maladaptive coping between the sexuality minoritised and heterosexual groups. However, this study employed a small sample, did not include gender minorities, and grouped sexual minorities together despite potential differences in outcomes within minority groupings (Beard et al., 2017; Rimes, Ion et al., 2019). No known adult or adolescent study has investigated outcomes in non-adapted DBT for gender minorities (see Harned et al., 2022 for review).

The current study, therefore, investigated clinical outcomes for SGM adolescents in a comprehensive DBT programme implemented in the UK National Health Service (NHS). This exploratory study presents data for specific sexual minority groups, rather than grouping all sexual minorities together, and for gender minority groups. Retrospective data was used from routine clinical practice. The aim is to provide practice-based outcome data for SGM adolescents, with the hope of stimulating and informing future studies. The exploratory research questions included:

1. What are the rates of treatment completion and non-completion for the different sexuality and gender groups within this DBT service?
2. Are there changes in clinical outcomes by the end of DBT for the different sexuality and gender groups within this service?
3. Does sexual orientation and gender identity group membership have an effect on clinical outcomes within this DBT service?

## Methods

### Ethics

Ethical approval was granted by the South London & Maudsley NHS Foundation Trust Clinical Governance (Audit and Service Evaluation) committee. Data were collected as part of routine service delivery.

### Design

This is a mixed independent and repeated measures design, with a sample stratified by sexual and gender identity (independent variables). The initial research question investigated the proportions who completed treatment (dependent variable) between sexual and gender identity groups in this service context (independent measures design). The second and third question explored changes in clinical outcomes (dependent variables; suicidal and NSSI behaviours, inpatient bed days, Accident and Emergency department (A&E) attendances, emotion dysregulation, reasons for living, and BPD, depression, and anxiety disorder symptoms) from a baseline time point compared to an end- or during-treatment time point (time points varied for some variables) for the sexual and gender identity groups within this service context (repeated measures design).

### Participants

The study inclusion criteria included being assessed as suitable for DBT pre-treatment for question 1 (*N* = 167), and starting and completing DBT treatment for question 2 (*N* = 112), over five years of service delivery. The inclusion criteria for the service included: 1) that participants were between 13 years and 17 years and four months (at referral; upper age limit varied depending on service capacity), 2) had engaged in at least one episode of self-injury in the past six months, and 3) had presented with symptoms in at least a further four domains of BPD as assessed according to the BPD subscale within the Structured Clinical Interview for DSM-V (SCID; First et al., 1997). While the young people needed only one episode of self-injury in the past six months to access the service, because this is a tier-4 setting most young people had high levels of suicidal behaviours and NSSI, which had typically not responded to lower-tiered interventions. The exclusion criteria for the service included a primary diagnosis of schizophrenia/psychosis, substance-use dependency, or another psychiatric disorder(s) that required more urgent assessment or treatment, or where the individual had opted out of the programme in the past three months.

### Treatment context

The National & Specialist CAMHS, DBT Service is a highly-specialist Tier 4 community DBT programme for adolescents (see Camp, Hunt, & Smith, 2023 and Smith et al., 2023 for further details on the programme). Tier 4 child and adolescent mental health services (CAMHS) in the UK are intensive and highly specialist interventions, which include specialist intensive outpatient treatment programmes, in-patient services, day programmes, and home intervention (NHS England, 2018). Tier 4 services are often only utilised when attempts to meet needs in lower-tiered services are unsuccessful. If young people met the service inclusion criteria, they were offered pre-treatment, comprising four to six sessions to learn about DBT, develop goals, and decide if this was the right time and treatment. If young people signed up to the DBT programme, they were offered eight to 12 months of treatment. Treatment included the four core modes of DBT (Camp, Hunt & Smith, 2023), informed the DBT model for adults (Linehan, 1993) and adolescents (Rathus, Miller, & Bonavitacola, 2018). Parents/carers were offered individual and family sessions as needed, alongside telephone coaching/support and a separate six-month-long parent/carer skills group informed by the DBT model for families (Fruzzetti, 2019).

All therapists attended weekly DBT consultation meetings (Linehan, 1993) and completed, as a minimum, foundation training in DBT via a licensed provider. Model adherence was supported by regular supervision, weekly team consultation meetings, and quarterly team consultation with an expert in DBT. As a principle-based treatment, some reasonable adaptations were made to treatment components to integrate identity- and stigma-related issues into participants’ treatment hierarchies and goals where relevant. Over the five years of service delivery covered within this evaluation, therapists experienced increasing training and awareness of SGM-associated difficulties. This included at least an annual teaching session on working with SGM young people and the contemporary published research in this area (e.g. Skerven et al., 2019). Exposure to these topics and an awareness of the high prevalence of SGM young people in the service resulted in increased use of sexuality- and gender-diverse examples in teaching, the disclosure of pronouns by therapists in skills groups, and the inclusion of socio-political invalidating environments within the teaching of the biosocial model. Additionally, therapists supported their clients with SGM-associated difficulties where they came up as links in chain analyses and where they were included in treatment goals. However, no fundamental adaptations were made to the skills modules or programme delivery with regards to SGM-related difficulties.

### Procedures

All data were collected via routine service delivery, with informed consent sought for treatment and questionnaire completion at intake from young people and their parents/carers. Demographic information was collected via a self-report questionnaire at assessment. Each question (with the exception of age, which offered a free text response option) was multiple choice, with an option for “prefer not to say” and “prefer to self-describe”. The question inquiring about sexual orientation was added six months into this service delivery period after clinical observations of the high prevalence of sexuality diverse adolescents accessing the service prompted the need for improved monitoring. Thus, there is higher missing data for this variable. Routine outcome measures (i.e. emotion dysregulation, BPD, depression, and anxiety symptoms, and reasons for living) were collected via Qualtrics^TM^ (Monash University, 2022) at assessment and at the end of treatment. Counts of NSSI and suicidal behaviours were collected for the first eight weeks of DBT (including pre-treatment) and final eight weeks of DBT using self-report on diary cards corroborated with reports from parents/carers and allocated professionals. Counts of A&E attendances and occupied inpatient bed days from the matched period before and during DBT were collected from NHS records and corroborated with reports from the young people, parents/carers, and professionals in their system.

## Measures

### The McLean Screening Instrument for Borderline Personality Disorder

(MSI-BPD) is a widely used screening instrument with good sensitivity and specificity for predicting the presence of BPD in adolescent populations (Zanarini et al., 2003). The 10 items are rated as “*no*” (0) or “*yes*” (1). Higher scores indicate higher presence of BPD symptoms and a score of 7 of above is the clinical cut-off (Zanarini et al., 2003). This measure’s validity and reliability have been demonstrated among adolescents (Noblin, Venta, & Sharp, 2013; Zanarini et al., 2003).

### The Difficulties in Emotion Regulation Scale

(DERS; Gratz & Roemer, 2004) is a self-report measure assessing multiple aspects of emotion dysregulation. The 36 items are measured on a five-point Likert scale from “*almost never*” (1) to “*almost always*” (5). Higher scores indicate more difficulty with emotion regulation and scores above 129 are suggested as a clinical cut-off (Camp, Hunt, & Smith, 2023). The DERS has good psychometric properties in adolescent populations (Gratz & Roemer, 2004; Neumann, van Lier, Gratz, & Koot, 2010; Weinberg & Klonsky, 2009).

### The Reasons for Living Inventory, Adolescent Version

(RFL; Osman et al., 1998), is a 32-item self-report measure of multiple protective factors against suicide. Items are rated on a scale from “*not at all important*” (1) to “*extremely important*” (6) and all items are averaged. Higher average scores indicate more protective factors for preventing suicidal behaviours. The RFL has good psychometric properties (Osman et al., 1998).

### The Moods and Feelings Questionnaire for Adolescents

(MFQ; Angold & Costello, 1987) is a self-report screening tool for depression in adolescents. The 33 items assess how young people have been feeling or acting recently, rated from “*not true*” (0) to “*true*” (2). Higher scores indicate higher severity of depression symptoms and scores above 28 are suggested as a clinical cut-off (Daviss et al., 2006). The MFQ has established psychometric properties (Angold & Costello, 1987; Daviss et al., 2006).

### The Screen for Child Anxiety-related Emotional Disorders for Adolescents

(SCARED; Birmaher et al., 1999) is a self-report measure assessing anxiety disorders in children and young people. The 41 items use a three-point Likert scale from “*not true or hardly ever true*” (0) to “*very true or often true*” (2). Total scores of 25 or above is suggested as the clinical cut-off (Birmaher et al., 1999). The SCARED has established psychometric properties (Birmaher et al., 1997; 1999).

**Occupied inpatient bed days** is a count of days, including an overnight stay, during which a young person is admitted as an inpatient due to concerns around mental health and risk.

**A&E attendances** is the count of attendances to the A&E department due to mental health crises, including but not limited to NSSI and suicidal behaviours.

**Self-injury** is the count frequency of NSSI and suicidal behaviours/incidents. Self-injury incidents are counted irrespective of motivation due to the difficulties differentiating based on motivation and intention (Bernegger et al., 2018; King, Cabarkapa, & Leow, 2019). Self-injury is defined as any intentional self-injury, such as cutting or burning skin, ingestion of harmful objects, or other methods to cause trauma to body tissue, irrespective of suicidal motivation (Bernegger et al., 2018; King et al., 2019).

## Data analysis

Data were analysed in SPSS Version 28 (IBM Corp, 2021). Missing data across clinical outcomes was 5.15% and missing at random (Little’s MCAR test: *X*^*2*^ = 328.49, *DF* = 327, *p* = .47). Missing outcome data were computed using expectation maximisation (Enders, 2001; Scheffer, 2002). In the overall sample there was no missing data for gender identity. Three percent in the overall sample endorsed the “prefer not to say” option for sexual orientation, which was treated as missing data. In the overall sample, after transforming “prefer not to say” into missing data, 13% had missing sexual orientation data (mostly due to this question being added to the questionnaire at a later date). Sexual orientations were grouped as bisexual/pansexual, gay/lesbian, and heterosexual to retain group size for statistical power while balancing not over generalising findings between sexuality minoritised groups. The gender identity groups were split into: 1) those whose gender identity was different from their sex assigned at birth (transgender) and 2) those whose gender identity and sex assigned at birth were the same (cisgender). Further disaggregated descriptive statistics are available in the Supplementary Materials.

Appropriate assumptions for each statistic test were checked. For question 1, Fisher’s Exact tests were used to explore proportions of treatment completion, as there were less than five counts within at least one group. For question 2, paired samples t-tests or Wilcoxon Signed Rank Test (WSRT; if t-test assumptions were not met) were used to investigate changes in outcomes from Time 1 to Time 2 for each group. Cohen’s (1988) *d* effect sizes for t-tests and Rosenthal’s (1994) *r* effect sizes for WSRTs were reported. For question 3, ANCOVA was used as a preliminary analysis investigating whether sexual orientation or gender identity group membership had an effect on end of treatment (or similar) outcomes, while controlling for baseline scores on the same measure. Partial eta-squared effect sizes were reported. Where the appropriate assumptions for the ANCOVA were not met, the Quade Nonparametric ANCOVA (Rae, 1985) was used instead.

## Results

### Descriptive Statistics

Sociodemographic data for the sample are presented in Table 1 and 2. In the overall sample, 65% identified as sexual minorities and 17% as gender minorities. These were not mutually exclusive and thus many sexual minorities identified as gender minorities and vice versa.

### Question 1. Treatment completion and non-completion

For the disaggregated sexual orientation groups: 33 (62%) of the bisexual group completed DBT, 12 (23%) opted out during DBT before completion, and eight (15%) opted out in pre-treatment before signing up to DBT; 13 (81%) of the pansexual group completed DBT, one (6%) opted out during DBT, and two (13%) opted out during pre-treatment; 15 (65%) of the gay/lesbian group completed treatment, two (9%) opted out in treatment, and six (26%) opted out during pre-treatment; and 36 (74%) of the heterosexual group completed DBT, seven (14%) opted out during treatment, and six (12%) opted out during pre-treatment. For the disaggregated gender identity groups: four (44%) of the binary transgender participants completed DBT, two (23%) opted out during treatment before completion, and three (33%) opted out in pre-treatment before signing up to the DBT programme; 15 (79%) of the non-binary participants completed DBT, two (10.50%) opted out in treatment, and two (10.50%) opted out in pre-treatment; and 93 (67%) of the cisgender participants completed treatment, 22 (16%) opted out during DBT, and 24 (17%) opted out during pre-treatment. The Fisher’s exact test found no significant differences in the proportion of young people who completed treatment and those who did not across the aggregated sexual orientation and gender identity groups (see Table 3).

### Question 2. Changes in clinical outcomes by the end of DBT

The paired samples t-tests and WSRT found a significant improvement in the majority of clinical outcomes from the start to the end of DBT for each group, with medium to large effect sizes (see Table 4 and 5). This was with the exception of the SCARED for heterosexual participants and self-injury for gay/lesbian participants, which were not significantly different by the end of treatment and had small effect sizes.

### Question 3. Differences in clinical outcomes by sexual orientation or gender identity

According to the ANCOVA models, after adjusting for baseline scores on the corresponding measures, there was no significant differences between sexual orientation groups at the end of DBT for scores on the MSI-BPD (*F*[2,93] = 0.28, *p* = .76, partial *η*^*2*^ = <.01), the DERS (*F*[2,93] = 0.40, *p* = .67, partial *η*^*2*^ = <.01), the MFQ (*F*[2,93] = 0.19, *p* = .83, partial *η*^*2*^ = <.01), and the SCARED (*F*[2,93] = 0.69, *p* = .51, partial *η*^*2*^ = .02). After adjusting for baselined RFL scores, there was an overall significant difference in end-treatment RFL scores between sexual orientation groups (*F*[2,93] = 3.31, *p* = .04, partial *η*^*2*^ = .07). However, post-hoc comparisons, with a Bonferroni correction, found no significant differences in RFL scores between the separate sexual orientation groups. However, there were emerging (non-significant) lower adjusted scores on the RFL for heterosexual participants compared to bisexual/pansexual (mean difference of -0.52, *p* = .10) and for the gay/lesbian group compared to the bisexual/pansexual group (mean difference of -0.67, *p* = .13). There was no emerging difference between the heterosexual and gay/lesbian group (mean difference of -0.15, *p* = 1.00).

According to the Quade Nonparametric Analysis of Covariance models, after adjusting for baseline rates of the corresponding variables, there was no significant differences between sexual orientation groups on the counts of self-injury incidents in the final eight weeks of DBT (*F*[2,94] = 1.37, *p* = .25) or A&E attendances during DBT (*F*[2,94] = 1.38, *p* = .26). After adjusting for before-DBT occupied inpatient bed days, there was a significant difference between sexual orientation groups in occupied inpatient bed days during DBT (*F*[2,94] = 4.86, *p* = .01). Post-hoc comparisons suggested that adjusted rates of inpatient bed days were higher for heterosexual participants compared to bisexual/pansexual participants (*t*[94] = 3.02, *p* = <.01). There was a (non-significant) trend towards the heterosexual participants having higher adjusted bed days in DBT compared to the gay/lesbian participants (*t*[94] = 1.95, *p* = .05). The comparison between the bisexual/pansexual and gay/lesbian groups were not statistically significant (*t*[94] = -0.24, *p* = .81).

According to the ANCOVA models, after adjusting for baseline scores on the corresponding measures, there was no significant differences between gender identity groups at the end of DBT for scores on the MSI-BPD (*F*[1,109] = 0.12, *p* = .73, partial *η*^*2*^ = <.01 5%POWER), the DERS (*F*[1,109] = 0.68, *p* = .41, partial *η*^*2*^ = <.01), the RFL (*F*[1,109] = 0.27, *p* = .60, partial *η*^*2*^ = <.01), the MFQ (*F*[1,109] = 0.95, *p* = .33, partial *η*^*2*^ = <.01), and the SCARED (*F*[1,109] = 0.40, *p* = .53, partial *η*^*2*^ = <.01). According to the Quade Nonparametric Analysis of Covariance models, after adjusting for baseline rates of the corresponding variables, there were no significant differences between gender identity groups on the count of self-injury incidents in the final eight weeks of DBT (*F*[1,110] = 0.01, *p* = .91), A&E attendances during DBT (*F*[1,110] = 0.02, *p* = .96), or occupied inpatient bed days during DBT (*F*[1,110] = 0.13, *p* = .72).

## Discussion

This study investigated clinical outcomes for adolescents with different sexual and gender identities in a highly-specialist CAMHS DBT programme. The overall number of young people completing the DBT programme after starting is similar to other evaluations of DBT for adolescents (42-88%; Johnstone et al., 2021). No significant differences between groups were observed in the proportions completing DBT. However, it is possible that any differences may not have been sufficiently powered in this small sample once stratified. This study opted to not collapse groups further where possible due to the variability in outcomes found between minoritised groups in other studies (e.g. Beard et al., 2017; Rimes, Ion et al., 2019). Nonetheless, it is of note that the completion rate for bisexual/pansexual and gay/lesbian groups were around 10% lower compared to the heterosexual group, and disaggregated data suggests this was even lower for bisexual groups. Additionally, while the differences between gender groups were minimal, the disaggregated binary transgender group, albeit small, had a completion rate of 44% compared to 67% and 79% for the cisgender and nonbinary groups, respectively. As these findings were not statistically significant, inferences about any potential differences cannot be made. However, completion rates require investigation in future research with larger sample sizes as this may suggest that there are aspects of DBT pre-treatment and treatment that are less acceptable to these groups, resulting in potentially higher treatment attrition. Barriers to the acceptability of DBT and similar interventions for SGM groups include worries about therapist-held judgements and stigma, concerns regarding confidentiality, a lack of therapist awareness of SGM-associated difficulties, and being unable to integrate SGM topics into therapy (Camp, Morris, et al., 2023; Hafeez et al., 2017; Pattinson et al., 2021).

SGM adolescents in this service, alongside their heterosexual and cisgender peers, appeared to experience reductions in self-injury, A&E attendance, inpatient service use, emotion dysregulation, and BPD, depression, and anxiety symptoms, as well as improvements in reasons for living. Sexual orientation and gender identity group membership did not appear to impact most clinical outcomes in this service after controlling for baseline symptoms. However, adjusted rates of occupied inpatient bed days during DBT were significantly higher for heterosexual participants compared to bisexual/pansexual groups. This is largely consistent with Poon et al.’s (2022) findings that sexuality minoritised adolescents had reductions in clinical outcomes in their DBT programme. It is of note that the pre-to-post effect sizes for the bisexual/pansexual and gay/lesbian groups were larger compared to the heterosexual group, as well as for the transgender group compared to the cisgender group. However, no significant change was detected in self-harm for the gay/lesbian group or anxiety symptoms for the heterosexual group, with small effect sizes. These findings suggest that there may be improvements by the end of this DBT programme in clinical outcomes for SGM groups, and that these measures may be sensitive to change to inform future studies. However, these findings are based on treatment-completers and need to be considered in the context the possible above-mentioned lower DBT completion rates for the SGM groups.

This DBT programme did not fundamentally adapt treatment delivery, beyond reasonable adaptions given to areas of need, for supporting SGM adolescents. However, it may be that increasing therapist awareness and knowledge of SGM-related difficulties increased their skills in supporting this group and integrating diversity into the modes of treatment (e.g. disclosing pronouns and using SGM examples in skills group), which may contribute towards consistent outcomes across groups. Indeed, SGM adolescents in this service have reported that there were strengths in how DBT therapists created safety and integrated SGM topics into therapy (Camp, Morris, et al., 2023). This does not seek to diminish the need for treatment adaptations for SGM-associated needs, such as those developed by Skerven et al. (2019). These culturally-responsive treatment offers represent useful approaches to reduce stigma and oppression in practice, and build in minority-specific needs, to reduce barriers to access and increase satisfaction. This is important given the research suggesting that SGM adolescents experiences barriers accessing and within services (e.g. Williams and Chapman, 2011), and that standard models such as DBT may not sufficiently meet the unique needs of SGM youth (Camp, Morris, et al., 2023). Instead, it may mean that standard DBT as delivered in this service context for adolescents is sufficiently flexible to be applied to some needs and distress of SGM young people to support with the reduction of *traditional* clinical outcomes.

An effective treatment targeting high-risk behaviours and emotion dysregulation may be particularly important for SGM adolescents given their higher risk of difficulties in these areas and to reduce the potential mediating effect of emotion dysregulation in their mental distress (Hatzebuehler, 2009; King et al., 2008; Liu et al., 2019). It is also of interest that this cohort of young people, which were assessed as suitable for a highly specialist DBT programme, contain a large representation of SGM individuals; 65% were sexual minorities and 17% gender minorities in the overall sample. This is significantly higher than the number of SGM individuals thought to be in the general population of adolescents in the UK and USA (around 3-15% for sexual minorities and 0.2-2.0% for gender minorities; Gallup, 2022; Office for National Statistics, 2021a, 2021b; UK Government Equalities Office, 2018). A higher representation of SGM adolescents in this service may in part represent the high prevalence of relevant difficulties in SGM young people. However, the proportions of SGM adolescents assessed as suitable for this DBT service (i.e. 7 to 85 times) remain higher than would be expected even when considering the increased prevalence rates of self-injury and suicidal behaviours (e.g. 1.5 to 7 times; Liu et al., 2019). This may suggest that there are particular SGM-associated needs not being met earlier in their treatment pathways and/or that they are experiencing barriers to accessing mental health services (e.g. clinician prejudice and misunderstanding; Hafeez et al., 2017; Pattinson et al., 2021). Thus, it may be that future research and implementation considers how to meet SGM adolescents’ needs earlier in their treatment trajectory or generally in mental health services.

This study has a number of limitations. Firstly, this study relies on self-report measures, which may be subject to bias in reporting. Attempts, where possible, to corroborate self-reported frequencies of self-injurious behaviours, and emergency and inpatient service use were made using national health service records, however due to the varied systems across different UK healthcare providers, it may be possible that count data are an under-representation. Secondly, no validated framework was used to collect self-injury data, which may decrease validity and replicability of this outcome. Thirdly, this study also presents outcome data from a sample of treatment completers, which likely represents the most optimistic version of results. However, an intention-to-treat analysis was not possible, due to difficulties obtaining end-treatment data from those who opted out before completion.

The sample was also recruited from a highly specialist national Tier 4 DBT programme, which local services only refer to once they have exhausted local options to target self-injury and suicidal behaviours. Therefore, this sample may constitute unique characteristics in regards to severity and complexity of need, which may not generalise to similar interventions in other service contexts. While attempts were made to split the sample by appropriate SGM subgroups due to the evidence showing heterogeneity of outcomes and experiences of each group (e.g. Rimes, Ion et al., 2019), it was not possible to split the sample by sex assigned at birth, which has also been shown to be associated with different outcomes for SGM groups (Rimes, Gooship et al., 2019), due to the very low representation of male assigned sex at birth participants. The relatively small samples of minoritised groups, where these were able to be stratified, also reduced statistical power. Finally, while DBT therapists are highly trained and supervised, and attend weekly consult meetings to maintain adherence and treatment quality, no formal measure of adherence was used consistently in this clinical setting in order to be included as a reassurance of treatment fidelity. In addition, while it is likely that culturally-sensitive changes to treatment delivery were made over time (see Camp, Morris, et al., 2023 for examples), these were not systematically monitored. Thus, it is not possible to know to which extent these adaptations impacted outcomes for the minoritised groups.

This study presents exploratory findings from a range of SGM groups within an adolescent population, from a naturalistic clinical setting, which included more SGM groups (i.e. gender minorities) and a larger sample, compared to the one similar evaluation (e.g. Poon et al., 2022). Similar studies in different settings, cultural contexts, and with a diverse range of participant characteristics are required. Future studies could also use the control afforded in research settings to conduct more methodologically robust research (e.g. randomised controlled trials) to investigate if DBT, in the current format, is efficacious for SGM adolescents and may benefit from including SGM-specific outcomes (e.g. internalised heterosexism). In addition, it is important to continue the work of culturally-responsive intervention adaptations in order to reduce wider health inequalities, barriers to accessing services, and poorer experiences when in services (e.g. Hafeez et al., 2017; Pattinson et al., 2021), alongside supporting existing service contexts to better meet the needs of minoritised populations.

## Conclusions

This exploratory study found statistically significant improvements in most clinical outcomes for SGM individuals and their cisgender and heterosexual peers within this DBT programme. There were no statistically significant differences in treatment completion, however emerging differences indicating possible lower completion by sexual orientation and gender minority subgroups that require further investigation. This highly specialist service context included a high representation of SGM youth, even when considering inflated risk of self-injury, which highlights an interesting area for future investigation. Preliminary effect sizes and results intend to inform future research in this area.

## Supplementary Material

Supplementary Material

## Figures and Tables

**Table. 1:** Sociodemographic Characteristics & Treatment Length by Sexual Orientation Groups

	Opted out in Pre-Treatment			Opted out in Treatment			Treatment Completers		
	Bisexual/Pansexual *(N* = 10)	Gay/Lesbian (*N* = 6)	Heterosexual (*N* = 6)	Bisexual/Pansexual (*N* = 13)	Gay/Lesbian (*N* = 2)	Heterosexual (*N* = 7)	Bisexual/Pansexual (*N* = 46)	Gay/Lesbian (*N* = 15)	Heterosexual (*N* = 36)
	*M (SD)*	*M* (*SD*)	*M* (*SD*)	*M* (*SD*)	*M* (*SD*)	*M* (*SD*)	*M* (*SD*)	*M* (*SD*)	*M* (*SD*)
Age	16.88 (0.94)	16.68 (1.09)	16.43 (0.76)	16.48 (0.97)	14.22 (0.14)	16.26 (0.79)	16.52 (1.02)	16.24 (1.00)	16.27 (1.00)
Treatment Length	-	-	-	4.83 (2.64)	7.00 (1.41)	4.83 (2.64)	10.46 (1.94)	10.73 (1.67)	10.77 (1.96)
	Freq (%)	Freq (%)	Freq (%)	Freq (%)	Freq (%)	Freq (%)	Freq (%)	Freq (%)	Freq (%)
Sex Assigned at Birth									
Female	10 (100%)	6 (100%)	6 (100%)	13 (100%)	2 (100%)	7 (100%)	46 (100%)	14 (93%)	35 (97%)
Male	0 (0%)	0 (0%)	0 (0%)	0 (0%)	0 (0%)	0 (0%)	0 (0%)	1 (7%)	1 (3%)
Gender Identity									
Binary Transgender	2 (20%)	0 (0|%)	1 (17%)	2 (15%)	0 (0%)	0 (0%)	1 (2%)	1 (7%)	1 (3%)
Non-binary	2 (20%)	0 (0%)	0 (0%)	0 (0%)	2 (100%)	0 (0%)	12 (26%)	3 (20%)	0 (0%)
Cisgender Female	6 (60%)	6 (100%)	5 (83%)	11 (85%)	0 (0%)	7 (100%)	33 (72%)	10 (66%)	34 (94%)
Cisgender Male	0 (0%)	0 (0%)	0 (0%)	0 (0%)	0 (0%)	0 (0%)	0 (0%)	1 (7%)	1 (3%)
Sexual Orientation									
Bisexual	8 (80%)	0 (0%)	0 (0%)	12 (92%)	0 (0%)	0 (0%)	32 (70%)	0 (0%)	0 (0%)
Pansexual	2 (20%)	0 (0%)	0 (0%)	1 (8%)	0 (0%)	0 (0%)	14 (31%)	0 (0%)	0 (0%)
Gay/Lesbian	0 (0%)	6 (100%)	0 (0%)	0 (0%)	2 (100%)	0 (0%)	0 (0%)	15 (100%)	0 (0%)
Heterosexual	0 (0%)	0 (0%)	6 (100%)	0 (0%)	0 (0%)	7 (100%)	0 (0%)	0 (0%)	36 (100%)
Ethnicity									
White British	4 (40%)	2 (33%)	5 (83%)	12 (92%)	2 (100%)	6 (86%)	30 (65%)	10 (67%)	26 (72%)
White Other	0 (0%)	2 (33%)	0 (0%)	0 (0%)	0 (0%)	1 (14%)	4 (9%)	2 (13%)	2 (6%)
Black/Black British	2 (20%)	2 (33%)	0 (0%)	0 (0%)	0 (0%)	0 (0%)	2 (4%)	1 (7%)	1 (3%)
Asian/Asian British	1 (10%)	0 (0%)	0 (0%)	1 (8%)	0 (0%)	0 (0%)	1 (3%)	0 (0%)	1 (3%)
Mixed Black/White	2 (20%)	0 (0%)	0 (0%)	0 (0%)	0 (0%)	0 (0%)	4 (9%)	0 (0%)	1 (3%)
Mixed Asian/White	0 (0%)	0 (0%)	0 (0%)	0 (0%)	0 (0%)	0 (0%)	3 (6%)	0 (0%)	2 (6%)
Other	1 (10%)	0 (0%)	1 (7%)	0 (0%)	0 (0%)	0 (0%)	2 (4%)	2 (13%)	3 (8%)

Note. *N* = sample size. Freq = frequency. *M* = mean. *SD* = standard deviation. Due to missing data on sexual orientation, sample size for sexual orientation treatment completers is smaller than for gender identity.

**Table. 2:** Sociodemographic Characteristics & Treatment Length by Gender Identity Groups

	Opted out in Pre-Treatment		Opted out in Treatment		Treatment Completers	
	Transgender^a^ (*N* = 5)	Cisgender *(N* = 24)	Transgender^a^ (*N* = 4)	Cisgender (*N* = 22)	Transgender^a^ (*N* = 19)	Cisgender (*N* = 93)
	*M (SD)*	*M (SD)*	*M (SD*)	*M (SD*)	*M (SD*)	*M (SD*)
Age	16.38 (1.17)	16.68 (0.82)	14.98 (1.24)	16.24 (1.08))	16.47 (1.01)	16.34 (0.99)
Treatment Length	-	-	5.00 (3.61)	4.53 (2.20)	10.89 (2.00)	10.66 (1.87)
	Freq (%)	Freq (%)	Freq (%)	Freq (%)	Freq (%)	Freq (%)
Sex Assigned at Birth						
Female	5 (100%)	24 (100%)	4 (100%)	22 (100%)	19 (100%)	89 (96%)
Male	0 (0%)	0 (0%)	0 (0%)	0 (0%)	0 (0%)	4 (4%)
Gender Identity						
Binary Transgender	3 (60%)	0 (0%)	2 (50%)	0 (0%)	4 (21%)	0 (0%)
Non-binary	2 (7%)	0 (0%)	2 (50%)	0 (0%)	15 (79%)	0 (0%)
Cisgender Female	0 (0%)	24 (100%)	0 (0%)	22 (100%)	0 (0%)	89 (96%)
Cisgender Male	0 (0%)	0 (0%)	0 (0%)	0 (0%)	0 (0%)	4 (4%)
Sexual Orientation						
Bisexual	2 (40%)	6 (35%)	1 (25%)	11 (61%)	8 (44%)	25 (32%)
Pansexual	2 (40%)	0 (0%)	1 (25%)	0 (0%)	5 (28%)	8 (10%)
Gay/Lesbian	0 (0%)	6 (35%)	2 (50%)	0 (0%)	4 (22%)	11 (14%)
Heterosexual	1 (20%)	5 (30%)	0 (0%)	7 (39%)	1 (6%)	35 (45%)
Ethnicity						
White British	3 (60%)	9 (41%)	4 (100%)	19 (90%)	16 (85%)	60 (65%)
White Other	0 (0%)	5 (23%)	0 (0%)	1 (5%)	1 (5%)	8 (9%)
Black/Black British	0 (0%)	4 (18%)	0 (0%)	0 (0%)	0 (0%)	4 (4%)
Asian/Asian British	1 (20%)	0 (0%)	0 (0%)	1 (5%)	1 (5%)	1 (1%)
Mixed Black/White	1 (20%)	1 (4%)	0 (0%)	0 (0%)	0 (0%)	8 (9%)
Mixed Asian/White	0 (0%)	0 (0%)	0 (0%)	0 (0%)	0 (0%)	6 (6%)
Other	0 (0%)	3 (14%)	0 (0%)	0 (0%)	1 (5%)	6 (6%)

Note. *N* = sample size. Freq = frequency. *M* = mean. *SD* = standard deviation.

a Inclusive of binary and non-binary transgender identities. Not all proportions total the same as the sample total due to missing data.

**Table. 3:** Treatment Completion and Non-Completion by Sexual Orientation and Gender Identity

	Opted out in Pre-	Opted out in	Completed	Fisher’s Exact
	Treatment	Treatment	Treatment	Test
	Freq (%)	Freq (%)	Freq (%)	
Sexual Orientation				
Bisexual/Pansexual	10 (15%)	13 (19%)	46 (67%)	
Gay/Lesbian	6 (26%)	2 (9%)	15 (65%)	5.19(ns)
Heterosexual	6 (12%)	7 (14%)	36 (74%)	
Gender Identity				
Transgender^a^	5 (17%)	4 (14%)	19 (68%)	0.09(ns)
Cisgender	24 (17%)	22 (16%)	93 (67%)	

Notes. Ns = not statistically significant at *p* = <.05.

a Inclusive of binary and non-binary transgender identities.

**Table. 4:** Inferential Statistics Comparing Pre- and Post-Treatment Clinical Outcomes for Sexual Orientation Groups

		Bisexual/Pansexual *(N =* 46)					Gay/Lesbian (*N* = 15)					Heterosexual (*N* = 36)			
Variable	Ax *M* *(SD)*	End *M* *(SD)*	*t*	*d*	95% *CI*	Ax *M* (*SD*)	End *M* (*SD*)	*t*	*d*	95% *CI*	Ax *M* (*SD*)	End *M* (*SD*)	*t*	*d*	95% *CI*
MSI-BPD	8.63 (1.42)	5.37 (3.33)	6.62**	**0.98**	0.62-1.35	8.93 (1.42)	5.60 (3.09)	4.71**	**1.22**	0.53-1.88	7.75 (2.10)	5.28 (3.49)	4.68**	**0.78**	0.40-1.15
DERS	138.26 (16.82)	107.76 (33.90)	5.92**	**0.87**	0.53-1.21	138.40 (22.10)	114.20 (31.07)	**3.46** **	**0.89**	0.28-1.49	137.68 (21.41)	113.04 (32.08)	4.26**	**0.71**	0.34-1.07
RFL	2.72 (0.89)	3.61 (1.08)	-4.56**	**-0.67**	-0.99-0.35	2.25 (0.72)	2.80 (0.60)	-2.35*	**-0.61**	-1.15-0.05	2.54 (1.09)	3.04 (1.29)	-2.35*	**-0.39**	-0.73-0.05
MFQ	45.67 (11.14)	34.77 (18.33)	3.83**	**0.56**	0.25-0.87	47.73 (8.89)	37.87 (15.29)	2.51*	**0.65**	0.08-1.20	45.33 (10.88)	36.72 (17.83)	3.49**	**0.58**	0.23-0.93
SCARED	49.24 (12.87)	42.38 (19.72)	**2.84** **	**0.42**	0.12-0.72	52.07 (17.34)	44.93 (19.25)	3.34**	**0.86**	0.56-1.45	50.18 (14.53)	46.85 (19.29)	1.39	0.23	-0.10-0.56
	Pre- *Mdn* (R)	Post- *Mdn* (R)	Z	*r*		Pre- *Mdn* (R)	Post- *Mdn* (R)	Z	*r*		Pre- *Mdn* (R)	Post- *Mdn* (R)	*Z*	*r*	
Self-Injury	3.00 (0-43)	0.00 (0-71)	-3.86**	**-0.57**		2.00 (0- 33)	0.00 (0- 42)	-1.50	-0.39		5.00 (0-56)	1.00 (0-11)	-4.07**	**-0.68**	
A&E	1.00 (0-20)	0.00 (0-10)	-3.93**	**-0.58**		2.00 (0- 10)	0.00 (0- 2)	-2.72**	**-0.70**		2.00 (0-15)	0.00 (0-5)	-3.22**	**-0.54**	
Inpatient Days	2.00 (0-341)	0.00 (0-2)	-4.31**	**-0.64**		0.00 (0- 335)	0.00 (0- 5)	-2.37*	**-0.61**		10.50 (0-180)	0.00 (0-14)	-4.06**	**-0.68**	

Notes. *p* = <.05, ** *p* = <.01. *N* = sample size. Ax = assessment, End = end of DBT. *M* = mean, *SD* = standard deviation. Cohen’s *d* small effect = ≥0.20, medium effect = ≥0.50, large effect = ≥0.80 (Cohen, 1988). Rosenthal’s *r* small effect = ≥0.10, medium effect = ≥0.30, large effect = ≥0.50 (Cohen, 1988). *T = paired samples t-test. Mdn* = median. *R* = range. *Z* = Wilcoxon Signed Rank Test *Z* statistic. MSI-BPD = MacLean Screening Instrument for BPD, clinical cut-off = ≥7. DERS = Difficulties with Emotion Regulation Scale, clinical cut-off = ≥128. RFL = Reasons for Living Inventory. MFQ = Moods and Feelings Questionnaire, clinical cut-off = ≥29. SCARED = Screen for Child Anxiety-Related Emotional Disorders, clinical cut-off = ≥25. Self-harm = count of suicidal and non-suicidal self-harm in the first eight weeks (Pre) and last eight weeks (Post) of DBT. A&E = count of Accident and Emergency Department visits in the matched period before DBT (Pre) and during DBT (Post). Inpatient Days = occupied inpatient bed days in the matched period before DBT (Pre) and during DBT (Post).

**Table. 5:** Inferential Statistics Comparing Pre- and Post-Treatment Clinical Outcomes for Gender Groups

Variable		Transgender *(N* = 19)					Cisgender (*N* = 93)			
	Ax *M (SD)*	End *M (SD)*	t	*d*	95% CI	Ax *M* (*SD*)	End *M* (*SD*)	*t*	*d*	*95% CI*
MSI-BPD	7.79 (2.02)	4.89 (3.21)	3.73**	**0.86**	0.32-1.38	8.54 (1.56)	5.70 (3.34)	8.82**	**0.92**	0.67-1.16
DERS	139.16 (22.50)	105.48 (31.26)	4.32**	**0.99**	0.43-1.53	136.47 (18.59)	110.81 (21.91)	7.58**	**0.79**	0.55-1.02
RFL	2.55 (0.94)	3.49 (1.12)	**-2.89** *	**-0.66**	-1.16-0.16	2.51 (1.01)	3.33 (1.16)	-6.11**	**-0.63**	-0.86-0.41
MFQ	46.89 (9.65)	32.52 (16.71)	3.33**	**0.76**	0.24-1.27	46.44 (10.47)	36.32 (17.52)	5.83**	**0.60**	0.38-0.82
SCARED	52.11 (14.12)	43.47 (18.88)	3.39**	**0.78**	0.25-1.29	49.80 (14.56)	43.75 (19.46)	3.85**	**0.40**	0.19-0.61
	Pre-*Mdn (R)*	Post-*Mdn* (*R*)	*Z*	*r*		Pre-*Mdn* (*R*)	Post-*Mdn* (*R*)	*Z*	*r*	
Self-Injury	4.00 (0-12)	0.00 (0-9)	-2.88**	**-0.66**		3.00 (0-56)	0.00 (0-71)	-5.48**	**-0.57**	
A&E	2.00 (1-15)	0.00 (0-10)	-2.42*	**-0.56**		1.00 (0-20)	0.00 (0-5)	-6.02**	**-0.62**	
Inpatient Days	2.00 (0-150)	0.00 (0-2)	-2.86**	**-0.66**		2.00 (0-341)	0.00 (0-17)	-6.10**	**-0.63**	

Notes. *p = <.05, **p = <.01. *N* = sample size. Ax = assessment, End = end of DBT. *M* = mean, *SD* = standard deviation. Cohen’s *d* small effect = ≥0.20, medium effect = ≥0.50, large effect = ≥0.80 (Cohen, 1988). Rosenthal’s *r* small effect = ≥0.10, medium effect = ≥0.30, large effect = ≥0.50 (Cohen, 1988). *t* = paired samples t-test. *Z* = Wilcoxon Signed Rank Test *Z* statistic. MSI-BPD = MacLean Screening Instrument for BPD, clinical cut-off = ≥7. DERS = Difficulties with Emotion Regulation Scale, clinical cut-off = ≥128. RFL = Reasons for Living Inventory. MFQ = Moods and Feelings Questionnaire, clinical cut-off = ≥29. SCARED = Screen for Child Anxiety-Related Emotional Disorders, clinical cut-off = ≥25. Self-harm = count of suicidal and non-suicidal self-harm in the first eight weeks (Pre) and last eight weeks (Post) of DBT. A&E = count of Accident and Emergency Department visits in the matched period before DBT (Pre) and during DBT (Post). Inpatient Days = occupied inpatient bed days in the matched period before DBT (Pre) and during DBT (Post).

## Data Availability

The data are not publicly available due to being considered sensitive clinical data due to being collected as part of routine service delivery.
