# Supplementary material for: Clinical Outcomes for Sexual and Gender Minority Adolescents in a Dialectical Behaviour Therapy Programme
Source: Behav Cogn Psychother. Author manuscript; Available in PMC 2024 Jul 11. (PMC7616180; doi:10.1017/S135246582400016X)
Supplement: Supplementary Material [file EMS194778-supplement-Supplementary_Materials.pdf]

Supplementary Material Table A.
Descriptive Statistics of Pre- and Post-Treatment Clinical Outcomes for Disaggregated Groups

	Bisexual (*N* = 33)		Pansexual (*N* = 13)		Binary Transgender (*N* = 4)		Nonbinary (*N* = 15)	
Variable	Ax *M* (*SD*)	End *M* (*SD*)	Ax *M* (*SD*)	End *M* (*SD*)	Ax *M* (*SD*)	End *M* (*SD*)	Ax *M* (*SD*)	End *M* (*SD*)
MSI-BPD	8.58 (1.42)	5.36 (3.43)	8.77 (1.48)	5.38 (3.18)	5.75 (2.22)	4.25 (2.63)	8.33 (1.63)	5.07 (3.41)
DERS	137.61 (18.24)	105.34 (34.85)	139.92 (13.00)	113.92 (31.83)	125.75 (34.19)	86.25 (31.21)	142.73 (18.31)	110.61 (30.23)
RFL	2.79 (0.80)	3.51 (1.08)	2.52 (1.11)	3.87 (1.07)	2.75 (1.14)	3.66 (1.34)	2.50 (0.92)	3.45 (1.10)
MFQ	45.30 (11.13)	32.57 (19.15)	46.62 (11.55)	40.34 (15.32)	41.00 (6.27)	24.75 (19.40)	48.47 (9.94)	34.59 (16.01)
SCARED	49.85 (12.07)	40.38 (20.85)	47.69 (15.15)	47.46 (16.14)	44.25 (15.37)	30.50 (24.34)	54.20 (13.54)	46.93 (16.44)
	Pre-*Mdn* (*R*)	Post-*Mdn* (*R*)	Pre-*Mdn* (*R*)	Post-*Mdn* (*R*)	Pre-*Mdn* (*R*)	Post-*Mdn* (*R*)	Pre-*Mdn* (*R*)	Post-*Mdn* (*R*)
Self-Harm	3.00 (0.00-32.00)	0.00 (0.00-9.00)	4.00 (0.00-43.00)	0.00 (0.00-71.00)	5.50 (2.00-8.00)	0.00 (0.00-3.00)	3.00 (0.00-12.00)	0.00 (0.00-9.00)
A&E	1.00 (0.00-20.00)	0.00 (0.00-7.00)	1.00 (0.00-10.00)	0.00 (0.00-10.00)	1.50 (1.00-2.00)	2.50 (0.00-10.00)	2.00 (1.00-15.00)	0.00 (0.00-7.00)
Inpatient Days	2.00 (0.00-341.00)	0.00 (0.00-2.00)	0.00 (0.00-302.00)	0.00 (0.00-0.00)	57.00 (0.00-150.00)	0.00 (0.00-1.00)	2.00 (0.00-147.00)	0.00 (0.00-2.00)

Notes. *N* = sample size. Ax = assessment, End = end of DBT. *M* = mean, *SD* = standard deviation. *Mdn* = median. *R* = range. MSI-BPD = MacLean Screening Instrument for BPD, clinical cut-off = ≥7. DERS = Difficulties with Emotion Regulation Scale, clinical cut-off = ≥128. RFL = Reasons for Living Inventory. MFQ = Moods and Feelings Questionnaire, clinical cut-off = ≥29. SCARED = Screen for Child Anxiety-Related Emotional Disorders, clinical cut-off = ≥25. Self-harm = count of suicidal and non-suicidal self-harm in the first eight weeks (Pre) and last eight weeks (Post) of DBT. A&E = count of Accident and Emergency Department visits in the matched period before DBT (Pre) and during DBT (Post). Inpatient Days = occupied inpatient bed days in the matched period before DBT (Pre) and during DBT (Post).
